# Analysis for discharge within 2 days after thoracoscopic anatomic lung cancer surgery

**DOI:** 10.1002/cam4.5982

**Published:** 2023-04-21

**Authors:** Yuanyuan Xu, Xiaoke Chen, Jianghao Ren, Mingyang Zhu, Ruonan Li, Jiazheng Huang, Yaxian Yao, Zhengmin Zhang, Qiang Tan

**Affiliations:** ^1^ Department of Thoracic Surgery, Shanghai Chest Hospital Shanghai Jiao Tong University Shanghai China

**Keywords:** early discharge, lung cancer, predictors, thoracoscopic surgery

## Abstract

**Objectives:**

The risk and beneficial factors of early discharge after thoracoscopic anatomic lung cancer surgery are unknown, and this study aims to investigate predictors and associated 30‐day readmission for early discharge.

**Methods:**

We performed a single‐center retrospective analysis of 10,834 consecutive patients who underwent thoracoscopic anatomic lung cancer surgery. Two groups were determined based on discharge date: “discharged by postoperative Day 2” and “discharged after postoperative Day 2.” Univariable and multivariable analysis were conducted to identify predictors for discharge. Using propensity score matching (PSM) to compare 30‐day readmission rate between two cohorts.

**Results:**

A total of 1911 patients were discharged by postoperative Day 2. Multivariable analysis identified older age (odds ratio (OR) = 1.014, *p* < 0.001), male sex (OR = 1.183, *p* = 0.003), larger tumor size (OR = 1.248, *p* < 0.001), pleural adhesions (OR = 1.638, *p* = 0.043), lymph nodes calcification (OR = 1.443, *p* = 0.009), advanced clinical T stage (vs. *T* < 2, OR = 1.470, *p* = 0.010), lobectomy resection (vs. segmentectomy resection, OR = 2.145, *p* < 0.001) and prolonged operative time (OR = 1.011, *p* < 0.001) as independent risk factors for discharge after postoperative Day 2. Three adjustable variables including higher FEV_1_/FVC (OR = 0.989, *p* = 0.001), general anesthesia (GA) plus thoracic paravertebral blockade (vs. GA alone, OR = 0.823, *p* = 0.006) and uni‐portal thoracoscopic surgery (vs. multi‐portal, OR = 0.349, *p* < 0.001) were associated with a decreased likelihood of discharge after postoperative Day 2. Before and after a 1:1 PSM, discharged by postoperative Day 2 did not increase the risk of 30‐day readmission compared to counterparts.

**Conclusions:**

Carefully selected patients can be safely discharged within 2 days after thoracoscopic anatomic lung cancer surgery. Three modifiable variables may be favorable for promoting discharge by postoperative Day 2.

## 1 INTRODUCTION

Enhanced recovery after surgery (ERAS) pathways have become a common approach in multiple surgical specialties, designed to reduce postoperative morbidity, improve pain control while minimizing opioid use, and promote early mobilization and recovery of bowel function with associated shorter hospital stays.^1^, ^2^, ^3^ An apparent benefit of ERAS pathways, followed by increased use of minimally invasive surgical procedures, has been widely used for anatomical lung cancer resection, significantly reducing length of hospital stay.^4^, ^5^, ^6^ Evolving management after thoracoscopic surgery has accelerated the discharge process for patients, some researches have even proposed a specific minimum hospitalization^7^, ^8^ and believe that this approach might be associated with improved outcomes.^8^, ^9^, ^10^

However, many thoracic surgeons remain reluctant to perform early discharge for fear that it may prevent adequate and timely detection and management of complications, leading to an increase in 30‐day readmission rate.^11^, ^12^ Moreover, readmission is a critically scrutinized indicator of the quality for primary care in hospitals and also associated with substantial costs that may offset any financial benefits gained from previously early discharge.^13^, ^14^ Therefore, it is important to carefully screen the clinical characteristics of patients suitable for early discharge and make comprehensive decisions to avoid readmission. To date, the literatures about early discharge following thoracoscopic anatomic lung resection were limited and most of them in absence of oncological data regarding tumor size, lymph node calcification or involvement and clinical tumor stage. In this study, by reviewing a large number of prospectively pooled data, including oncological factors, we aimed to explore the risk and favorable factors associated with early discharge to guide clinical practice.

## 2 MATERIALS AND METHODS

### 2.1 Study design and patients

**FIGURE 1:**
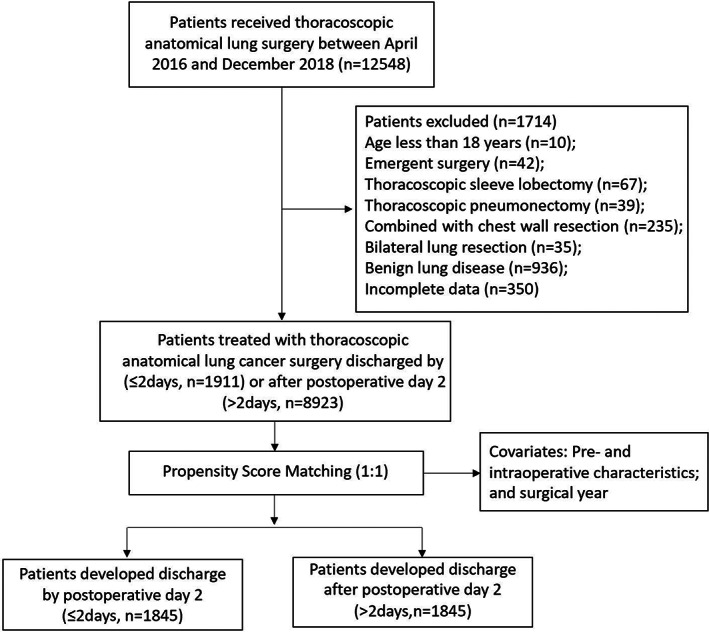
Patient flowchart.

After obtaining approval from the Institutional Review Board (IRB) of Shanghai Chest Hospital (IS2179), we performed a single‐institute retrospective study based on a prospectively collected database from April 2016 to December 2018, including 12,548 consecutive patients who underwent thoracoscopic anatomical lung cancer surgery. Excluded patients were listed in the flow diagram (Figure 1). A total of 10,834 patients were enrolled in the final analysis.

### 2.2 Anesthesia protocol

All patients were routinely monitored by electrocardiogram, pulse oximetry, non‐invasive blood pressure and capnography. Radial artery intubation and right internal jugular central venous catheterization were used to monitor invasive blood pressure. For thoracic paravertebral blockade (TPVB), 20 mL 0.5% ropivacaine was injected to the T4–T5 by the experienced anesthesiologist under the guidance of ultrasound before surgery. The intraoperative lung protective ventilation strategies consisted of low‐tide ventilation based on ideal body weight (≤8 mL/kg), PEEP = 5 cm H_2_O, lung recruitment and maintenance of airway pressure < 30 cm H_2_O. Extubation was performed in the operating room for all suitable patients. All patients received patient‐controlled analgesia pump, including sufentanil 1.0 μg/kg + desoxocin 0.4 mg/kg.

### 2.3 Technique of operation

Since 2016, the high‐volume center of Shanghai Chest Hospital has completed nearly 10,000 lung operations each year, of which thoracoscopic surgery accounts for more than 80 percent. A total of 12 physicians served as chief surgeons, in charge of 12 surgical groups, and each group also had one to two surgical assistants. All possible thoracoscopic surgical procedures (including uni‐ and multi‐portal) were determined by the participating surgeons based on the patient's preoperative evaluation, operative planning and surgical experience. Surgical experience was categorized into two groups (<300/year vs. ≥300/year), and the surgeons performed about 328 cases annually on average. For all patients, anatomic lung resection plus systematic lymph node dissection was regarded as the optimal treatment for lung cancer.

### 2.4 Data collection and definition

**FIGURE 2:**
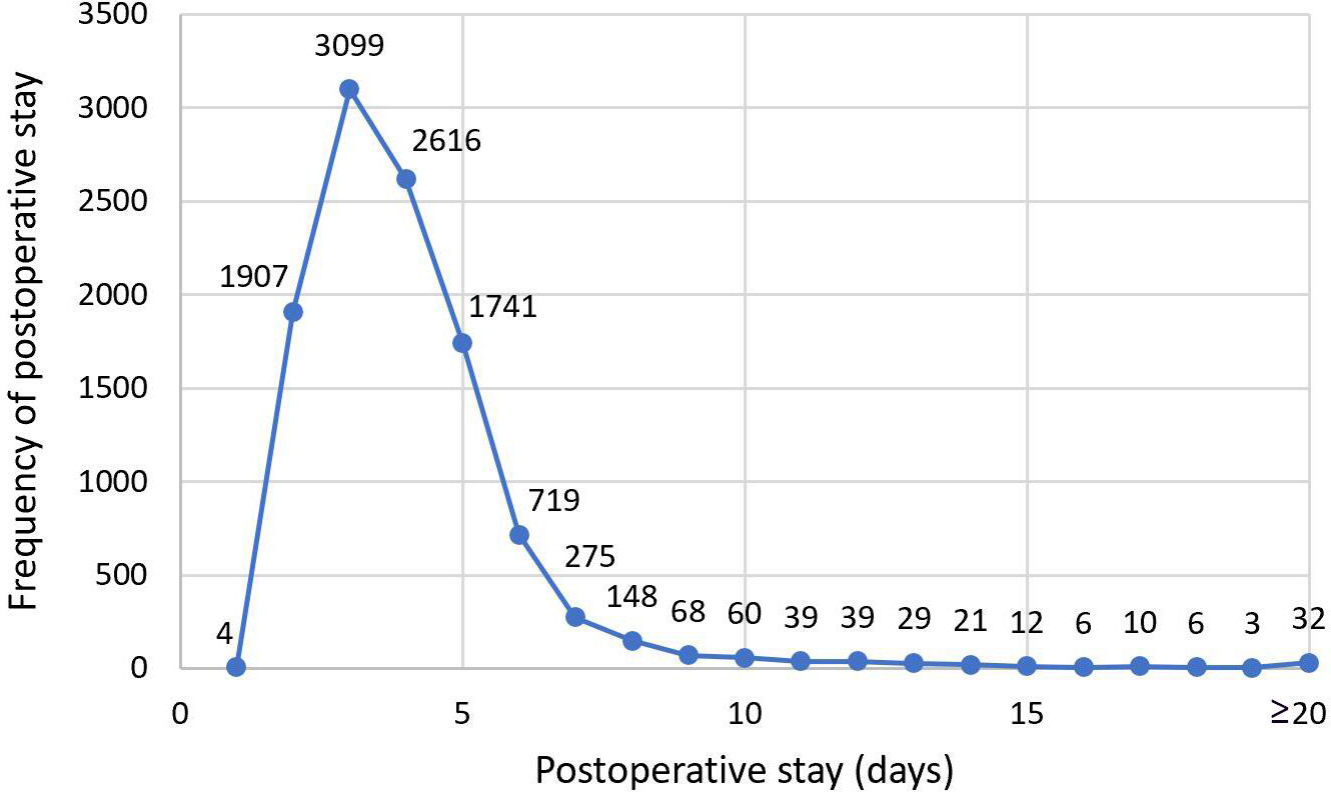
Description of postoperative stay.

Perioperative clinical data were prospectively extracted from our institution's electronic medical system, including patient's preoperative characteristics, surgical procedures, clinical tumor stage, postoperative complications, and 30‐day readmission. Based on the peak value of discharge date was 3 days (Figure 2) and the interquartile range (IQR) of postoperative stay was 3–5 days, patients were stratified into the two groups: “discharged by postoperative Day 2” and “discharged after postoperative Day 2.” Discharge was decided by the chief surgeon after taking careful consideration for a patient's pain, mobility, self‐care, respiratory status, oral intake and individual intention. Commonly, the chief surgeon adhered to relatively uniform discharge criteria, as follows: the patient had no or mild air leakage before discharge, no atelectasis, no fever, a chest drainage of <250 mL/day, and no abnormality in chest radiograph. The 30‐day readmission was defined as an unplanned return to the hospital due to various postoperative complications.

### 2.5 Statistical analysis

Continuous variables were compared between the two groups using Two independent sample *t*‐test or Mann–Whitney U test. Categorical variables were compared with Chi‐square test or Fisher exact test, depending on the sample size. Univariable analysis showed that all the factors significantly associated with discharge within 2 days (*p* < 0.2) were inserted into the multivariable logistic regression model using the forward selection strategy with LR. To reduce the selection bias and other potential confounding effects, we performed a 1:1 propensity score matching (PSM) analysis using a caliper size of 0.01 to compare the risk of 30‐day readmission rate between two cohorts. All pre‐, intraoperative variables and surgical year were included in the PSM. Standardized mean difference (SMD) between two cohorts on all covariables before and after matching was calculated, with differences of <10% indicating adequate balance in the cohort. Statistical analysis was performed using the SPSS 26.0 software (IBM Corp., Armonk). R version 4.1.2 was used using the forestplot, tidyr, dplyr, tableone, ggplot2, reshape2, survey and Matching packages. *p* < 0.05 was considered statistically significant.

## 3 RESULTS

### 3.1 Study cohort

From April 2016 to December 2018, 10,834 patients underwent thoracoscopic anatomical lung cancer surgery, of which 22.2% (2402 out of 10,834) underwent segmentectomy resection and 77.8% (8432 out of 10,834) underwent lobectomy resection, and 17.6% (1911 out of 10,834) developed discharge by postoperative Day 2 (Figure 1). The occurrence of discharge by postoperative Day 2 increased from 365 out of 3229 (11.3%), to 749 out of 3923 (19.1%), to 797 out of 3682 (21.6%) (*p* < 0.001) over 3‐year intervals.

### 3.2 Predictors for discharge by postoperative Day 2

**FIGURE 3:**
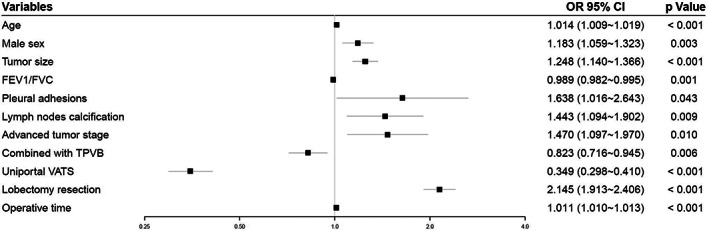
Forest plot for discharge after postoperative Day 2. FEV_1_, forced expiratory volume in 1 s; FVC, forced vital capacity; TPVB, thoracic paravertebral blockade; VATS, video‐assisted thoracoscopic surgery.

Univariable analysis identified that 19 variables were significantly associated with discharge after postoperative Day 2 (Tables 1 and 2). Multivariable analysis revealed that older age (odds ratio (OR) = 1.014, 95%CI, 1.009–1.019, *p* < 0.001), male sex (OR = 1.183, 95%CI, 1.059–1.323, *p* = 0.003), larger tumor size (OR = 1.248, 95%CI, 1.140–1.366, *p* < 0.001), pleural adhesions (OR = 1.638, 95%CI, 1.016–2.643, *p* = 0.043), lymph nodes calcification (OR = 1.443, 95%CI, 1.094–1.902, *p* = 0.009), advanced clinical tumor stage (vs. T < 2, OR = 1.470, 95%CI, 1.097–1.970, *p* = 0.010), lobectomy resection (vs. segmentectomy resection, OR = 2.145, 95%CI, 1.913–2.406, *p* < 0.001) and prolonged operative time (OR = 1.011, 95%CI, 1.010–1.013, *p* < 0.001) were independent risk factors for discharge after postoperative Day 2 (Figure 3). Three adjustable variables, including higher FEV_1_/FVC (OR = 0.989, 95%CI, 0.982–0.995, *p* = 0.001), general anesthesia (GA) plus TPVB (vs. GA alone, OR = 0.823, 95%CI, 0.716–0.945, *p* = 0.006) and uni‐portal thoracoscopic surgery (vs. multi‐portal, OR = 0.349, 95%CI, 0.298–0.410, *p* < 0.001), were associated with a decreased likelihood of discharge after postoperative Day 2 (Figure 3).

**TABLE 1:** Preoperative characteristics stratified by discharge date.

Variables^a^	POD ≤2 days (*n* = 1911)	POD >2 days (*n* = 8923)	SMD	*p* value
Age, years	55.3 ± 11.0	58.2 ± 10.5	0.272	<0.001^b^
Sex			0.186	<0.001^b^
Male sex	607 (31.8)	3628 (40.7)		
Female sex	1304 (68.2)	5295 (59.3)		
BMI, kg/m^2^	23.2 ± 2.9	23.3 ± 3.0	0.040	0.120
ASA grade			0.030	0.466
I	160 (8.4)	792 (8.9)		
II	1546 (80.9)	7247 (81.2)		
III/IV	205 (10.7)	884 (9.9)		
Comorbidity				
Hypertension	110 (5.8)	623 (7.0)	0.050	0.053
Diabetes mellitus	47 (2.5)	310 (3.5)	0.060	0.024^b^
Coronary artery disease	6 (0.3)	50 (0.6)	0.037	0.173
Stroke/TIA	4 (0.2)	28 (0.3)	0.020	0.445
FEV_1_/FVC, %	101.9 ± 7.3	101.0 ± 8.5	0.123	<0.001^b^
DLCO%	95.3 ± 15.8	94.3 ± 16.8	0.058	0.018^b^
Chemoradiotherapy	0 (0.0)	19 (0.2)	0.065	0.062
Tumor size, cm	1.5 ± 0.9	1.8 ± 1.0	0.314	<0.001^b^
Clinical tumor stage			0.337	<0.001^b^
T1a	769 (40.2)	2428 (27.2)		
T1b	793 (41.5)	3812 (42.7)		
T1c	231 (12.1)	1762 (19.7)		
T2a	71 (3.7)	592 (6.6)		
T2b	34 (1.8)	203 (2.3)		
T3	11 (0.6)	98 (1.1)		
T4	2 (0.1)	28 (0.3)		
Advanced clinical stage (T ≥ 2)	118 (6.2)	921 (10.3)	0.151	<0.001^b^
Surgical experience <300/year	533 (27.9)	2631 (29.5)	0.035	0.164

Abbreviations: ASA, american society of anesthesiology; BMI, body mass index; DLCO, diffusion capacity for carbon monoxide.FEV_1_, forced expiratory volume in 1 second; FVC, forced vital capacity; POD, postoperative day; SMD, standardized mean difference; TIA, transient cerebral ischemic attack.

^a^ Continuous data are shown as mean ± standard deviation and categoric data as number (%).

^b^ Statistically significant (*p* < 0.05).

**TABLE 2:** Intraoperative characteristics stratified by discharge date.

Variables^a^	POD **≤**2 days (*n* = 1911)	POD >2 days (*n* = 8923)	SMD	*p* Value
Lymph nodes calcification	72 (3.8)	435 (4.9)	0.054	0.038^b^
Clinical nodal involvement	49 (2.6)	454 (5.1)	0.132	<0.001^b^
Pleural adhesions	20 (1.0)	217 (2.4)	0.106	<0.001^b^
Type of resection			0.420	<0.001^b^
Segmentectomy resection	715 (37.4)	1687 (18.9)		
Lobectomy resection	1196 (62.6)	7236 (81.1)		
Procedure			0.233	<0.001^b^
Uni‐portal	278 (14.5)	653 (7.3)		
Multi‐portal	1633 (85.5)	8270 (92.7)		
Approach			0.177	<0.001^b^
VATS	1886 (98.7)	8549 (95.8)		
RATS	25 (1.3)	374 (4.2)		
Anesthesia type			0.078	0.001^b^
General anesthesia	1582 (82.8)	7642 (85.6)		
Combined with TPVB	329 (17.2)	1281 (14.4)		
Location of resection			0.024	<0.001^b^
Left resection	712 (37.3)	3428 (38.4)		
Right resection	1199 (62.7)	5495 (61.6)		
Ipsilateral reoperation	1 (0.1)	12 (0.1)	0.027	0.487
Conversion to thoracotomy	8 (0.4)	125 (1.4)	0.104	<0.001^b^
Intraoperative transfusion	0 (0)	26 (0.3)	0.076	0.016^b^
Intraoperative new onset AF	16 (0.8)	153 (1.7)	0.078	0.005^b^
Operative time, min	85.8 ± 32.3	99.3 ± 36.5	0.393	<0.001^b^

Abbreviations: AF, atrial fibrillation; POD, postoperative day; RATS, robotic‐assisted thoracoscopic surgery; SMD, standardized mean difference; TPVB, thoracic paravertebral blockade; VATS, video‐assisted thoracoscopic surgery.

^a^ Continuous data are shown as mean ± standard deviation and categoric data as number (%).

^b^ Statistically significant (*p* < 0.05).

### 3.3 Outcomes

The outcomes between two groups were described in Table S1, and showed that discharge after postoperative Day 2 was associated with higher rates of postoperative pulmonary complications, arrhythmia and transfusion (All *p* < 0.001). The risk of 30‐day readmission was similar in patients discharged by postoperative Day 2 compared to patients discharged after postoperative Day 2 (Table S2). After a 1:1 PSM analysis, all patient pre‐, intraoperative variables and surgical year were comparable among two cohorts (Table S3A,B). We investigated outcomes in 3690 patients (1845 pairs) and found that discharged by postoperative Day 2 did not increase the risk of 30‐day readmission compared to counterparts (Table S4).

### 3.4 Causes of 30‐day unplanned readmission

**FIGURE 4:**
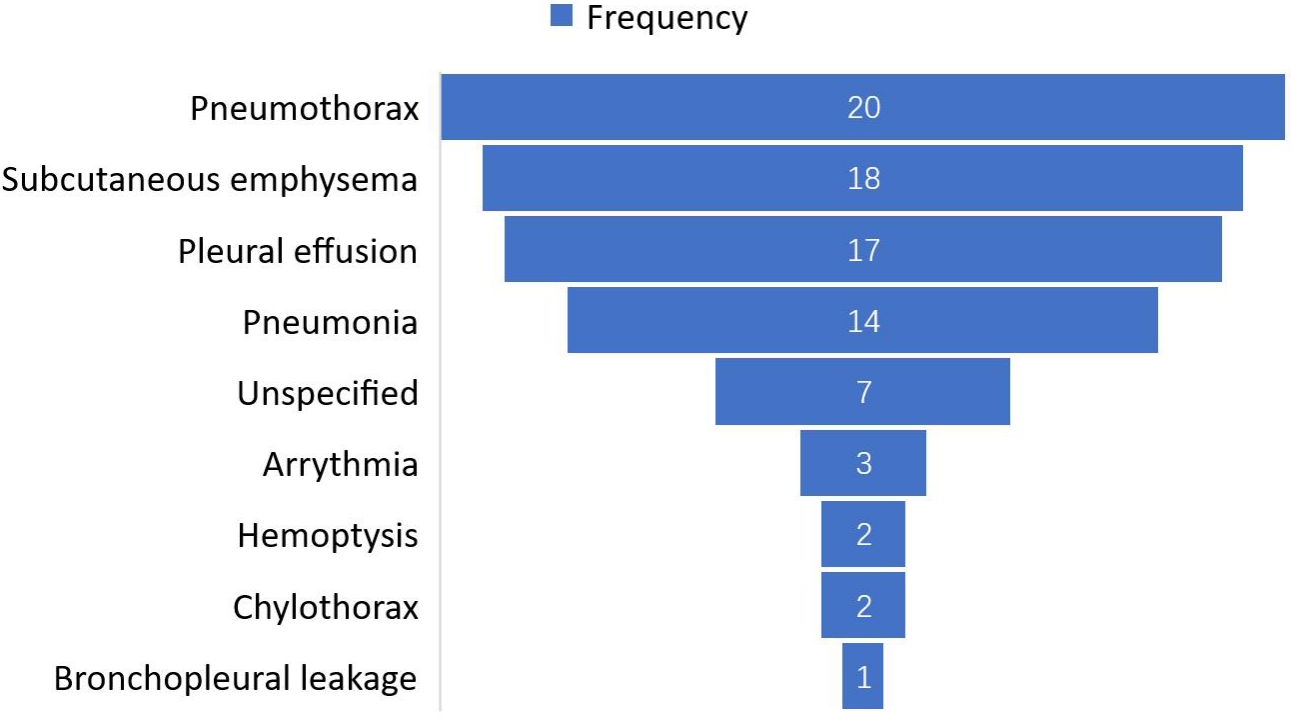
Frequency and causes of 30‐day readmission.

A total of 84 patients were readmitted within 30 days. The most common cause was pneumothorax, found in 20 patients (23.8%), followed by subcutaneous emphysema in 18 (21.4%), pleural effusion in 17 (20.2%) and pneumonia in 14 (16.7%). Other causes leading to 30‐day readmission, including arrythmia (3.6%), hemoptysis (2.4%), chylothorax (2.4%), and bronchopleural leakage (1.2%). Moreover, detailed reasons for 30‐day readmission were not recorded for seven patients in the study due to incomplete data (Figure 4).

### 3.5 Comments

In total, 1911 patients developed discharge within 2 days after thoracoscopic anatomical lung cancer surgery. The rate of unplanned 30‐day readmission was 0.78% and its main leading causes was pneumothorax. Our research also identified eight independent risk factors and three favorable factors for discharge after postoperative Day 2. There was no sufficient evidence that discharge by postoperative Day 2 was associated with an increased rate of unplanned 30‐day readmission after surgery.

Early discharge has different definitions and criteria in various documents, without a specific minimum postoperative stay.^8^, ^9^, ^10^, ^11^, ^15^, ^16^ Currently, the most frequently used date of early discharge was 2 days or 1 day after surgery. In our study, patients were classified into two groups at discharge by postoperative Day 2 based on the peak value of discharge date was 3 days and the IQR of postoperative stay was 3–5 days. Besides, by reviewing the postoperative data of 10,834 patients in this study, only four patients were discharged on the first day after surgery. Therefore, it is not appropriate for this study to define early discharge as discharge on postoperative Day 1.

Many attempts have been made to identify underlying risk factors for early discharge. And these studies have demonstrated that increasing age, male sex, higher BMI, and ASA grade, smokers, history of dyspnea, lower FEV_1_% predicted, DLCO < 60%, thoracotomy, prolonged operative time and use of multiple chest tube or epidural catheter all decreased the likelihood of early discharge.^11^, ^15^, ^16^, ^17^, ^18^, ^19^ By comparison, our study also verified that older age, male sex, type of resection, and prolonged operative time were independent risk factors for discharge after postoperative Day 2. In addition, other potential predictors for discharge after postoperative Day 2 were explored, including large tumor size, pleural adhesions, lymph nodes calcification and advanced clinical tumor stage. Meanwhile, three modifiable beneficial factors rarely reported for discharge by postoperative Day 2 included higher FEV_1_/FVC, uni‐portal VATS, and GA combined with TPVB.

Elderly patients have reduced pulmonary function reserves, increased comorbidities and tissue fragility,^20^ which may be associated with increased complications as well as length of hospital stay. The occurrence of male sex discharged after postoperative Day 2 was higher with no clear biological explanation. And this relationship may not be directly causal, but a possible explanation was the higher incidence of postoperative poor outcomes.^21^, ^22^ In addition, extensive lung resection was a marker of complexity or difficulty and might increase operative time and perioperative adverse complications,^23^, ^24^ where methods to reduce postoperative air leakage, such as fissure‐less dissection, in turn, were also likely beneficial in reducing length of hospital stay.^25^

It is well known that thoracoscopic anatomic lung resection combined with systematic lymph node removal is regarded as the optimal treatment for lung cancer. In this cohort study, we elucidated the relationship between tumor size, lymph node calcification and advanced clinical tumor stage and discharge by postoperative Day 2. As we speculated, to some extent, all three variables reduced the likelihood of discharge by postoperative Day 2. Likewise, pleural adhesion increased the difficulty of lung tissue resection and conversion rates,^23^ leading to prolonged operative time, and was associated with a longer postoperative hospital stay. Thus, ERAS pathways should be personalized to specific expectations, such that patients at higher risk of perioperative events may receive specialized preventive care.

In addition, our study explored three possible predictors that may benefit patients from discharge by postoperative Day 2, providing an effective approach for clinical practice. Previous studies have indicated the predictive role of preoperative pulmonary function assessment (including FEV_1_% predicted, DLCO < 60%) in early discharge,^15^, ^18^ our study also showed the value of preoperative FEV_1_/FVC in predicting discharge by postoperative Day 2. Pain control was also a factor affecting length of hospital stay, and difficulty in perioperative pain control may be a barrier to early discharge after surgery.^26^ Our research confirmed that the application of TPVB may accelerate the process of discharge, possibly due to the reduction of intraoperative surgical stress, especially in dissection of mediastinal and hilar lymph nodes, and the decrease of postoperative pain and associated inflammatory responses.^27^, ^28^ Uni‐portal VATS was another feasible procedure to promote discharge by postoperative Day 2, which may be related to alleviating postoperative pain and promoting earlier mobilization.^26^, ^29^, ^30^

In terms of 30‐day readmission, the risk of occurrence between two groups before and after a 1:1 PSM was not statistically significant. Linden et al conducted a retrospective cohort study based on the Society of Thoracic Surgeons General Thoracic Surgery Database (STS‐GTSD) and found that patients receiving anatomic lung resection discharged on Day 1 were not at increased risk of readmission and mortality.^18^ Forster et al. also showed no difference in 30‐day readmission between patients discharged within 3 days and those discharged after 3 days in thoracoscopic anatomic lung cancer surgery.^15^ Even among patients treated with the enhanced recovery pathway, Chevrollieret al. concluded that discharge within 2 days after minimally invasive anatomic lung resection did not increase the risk of readmission.^10^

This retrospective study has several limitations. First, as a retrospective study based on prospectively collected data, it has the inherent design biases. Second, due to the limited granularity of postoperative care data, details of which criteria and cutoffs are essential for safe postoperative Day 2 discharge cannot be quantified by this study. Also, this study included several groups of surgeons whose preferences and techniques may have varied over time. Third, the relationship between discharge by postoperative Day 2 and patients' long‐term outcomes needs further investigation.

## 4 CONCLUSIONS

By conducting a monocentric retrospective analysis of 10,834 patients receiving thoracoscopic anatomic lung cancer surgery, there was insufficient evidence that discharge by postoperative Day 2 could increase the risk of 30‐day unplanned readmission. This study also identified 11 independent predictors for discharge after postoperative Day 2, among these including three modifiable variables which may promote suitable patients safely discharged by postoperative Day 2.

## AUTHOR CONTRIBUTIONS

**Yuanyuan Xu:** Formal analysis (equal); investigation (equal); methodology (equal); resources (equal); writing – original draft (equal); writing – review and editing (equal). **Xiaoke Chen:** Data curation (equal); formal analysis (equal); investigation (equal); methodology (equal); resources (equal); writing – original draft (equal); writing – review and editing (equal). **Jianghao Ren:** Formal analysis (equal); investigation (equal); methodology (equal); resources (equal); writing – original draft (equal); writing – review and editing (equal). **Mingyang Zhu:** Software (equal); supervision (equal); validation (equal); visualization (equal); writing – original draft (equal); writing – review and editing (equal). **Ruonan Li:** Software (equal); supervision (equal); validation (equal); visualization (equal); writing – original draft (equal); writing – review and editing (equal). **Jiazheng Huang:** Software (equal); supervision (equal); validation (equal); visualization (equal); writing – original draft (equal); writing – review and editing (equal). **Yaxian Yao:** Software (equal); supervision (equal); validation (equal); visualization (equal); writing – original draft (equal); writing – review and editing (equal). **Zhengmin Zhang:** Conceptualization (equal); data curation (equal); funding acquisition (equal); project administration (equal); writing – original draft (equal); writing – review and editing (equal). **Qiang Tan:** Conceptualization (equal); data curation (equal); funding acquisition (equal); project administration (equal); writing – original draft (equal); writing – review and editing (equal).

## FUNDING INFORMATION

This work was supported by National Natural Science Foundation of China (81871497), Shanghai Chest Hospital Basic Research Institute Cultivation Project (2019YNJCQ06), Science and Technology Innovation Special Fund of Shanghai Jiao Tong University (ZH2018QNA65).

## CONFLICT OF INTEREST STATEMENT

All authors have no conflicts of interest to declare.

## ETHICS STATEMENT

This study was approved by the Institutional Review Board at Shanghai Chest Hospital (IS2179), and the informed consent was waived because of the retrospective nature of the study.

## Supporting information


Table S1.
**Table S2**.
**Table S3a**.
**sTable S3B**.
**Table S4**.

## Data Availability

Our research team could provide original data under reasonable request and with permission from Shanghai Chest Hospital.
