# Supplementary material for: Analysis for discharge within 2 days after thoracoscopic anatomic lung cancer surgery
Source: Cancer Med. 2023 Apr 21;12(12):13054–62. doi: 10.1002/cam4.5982 (PMC10315791; doi:10.1002/cam4.5982)
Supplement: Supplementary file 1 — Table S1. Table S2. Table S3a. sTable S3B. Table S4. [file CAM4-12-13054-s001.docx]

Supplementary **table S1** **Description of outcomes based on discharge date**

| Outcomes | POD≤2days  (n=1911) | POD>2days  (n=8923) | *P* Value |
| --- | --- | --- | --- |
| Length of stay, day | 2(2-2) | 4(3-5) | <0.001^b^ |
| PPCs | 14(0.7) | 3663(41.1) | <0.001^b^ |
| Postoperative arrhythmia | 16(0.8) | 277(3.1) | <0.001^b^ |
| Postoperative transfusion | 2(0.1) | 76(0.9) | <0.001^b^ |
| 30-day TIA or stroke | 1(0.05) | 25(0.28) | 0.071 |
| 30-day readmission | 11(0.6) | 73(0.8) | 0.273 |
| 30-day mortality | 0(0) | 2(0.022) | 1.000 |

Length of stay presented as median (IQR) and remaining data presented as number (%). PPCs: Postoperative pulmonary complications; TIA: Transient cerebral ischemic attack.

Supplementary table S2 Multivariable **logistic regression analysis** of predictors for 30-day readmission

| Variables | OR (95% CI) | *P* Value | |
| --- | --- | --- | --- |
| Age, years | 1.004 (0.981~1.028) | | 0.713 |
| Male sex | 1.501 (0.932~2.417) | | 0.095 |
| BMI, kg/m^2^ | 0.952 (0.877~1.033) | | 0.240 |
| ASA grade |  | |  |
| I(reference) |  | |  |
| II | 0.495 (0.262~0.934) | | 0.030^b^ |
| III/IV | 0.275 (0.094~0.802) | | 0.018^b^ |
| Hypertension | 1.002 (0.391~2.570) | | 0.997 |
| Coronary artery disease | 2.807 (0.352~22.396) | | 0.330 |
| Stroke/TIA | 5.661 (0.708~45.294) | | 0.102 |
| FEV_1_/FVC, % | 0.974 (0.951~0.998) | | 0.033^b^ |
| DLCO% | 1.001 (0.988~1.014) | | 0.917 |
| Chemoradiotherapy | 2.896 (0.286~29.370) | | 0.368 |
| Tumor size, cm | 1.140 (0.856~1.517) | | 0.370 |
| Advanced clinical stage (T≥2) | 0.966 (0.389~2.399) | | 0.941 |
| Surgical experience < 300/y | 1.082 (0.641~1.828) | | 0.767 |
| Lymph nodes calcification | 1.850 (0.891~3.840) | | 0.099 |
| Clinical nodal involvement | 2.186 (1.069~4.470) | | 0.032^b^ |
| Lobectomy resection | 1.696 (0.813~3.535) | | 0.159 |
| Multi-portal | 1.081 (0.457~2.552) | | 0.860 |
| RATS | 3.354 (1.708~6.589) | <0.001^b^ | |
| Combined with TPVB | 1.701 (0.958~3.021) | | 0.070 |
| Right resection | 1.032 (0.651~1.637) | | 0.893 |
| Conversion to thoracotomy | 1.992 (0.549~7.222) | | 0.295 |
| Intraoperative new onset AF | 0.566 (0.076~4.197) | | 0.577 |
| Operative time, mins | 1.000 (0.994~1.007) | | 0.938 |
| PPCs | 0.694 (0.415~1.161) | | 0.164 |
| Postoperative arrhythmia | 0.664 (0.152~2.907) | | 0.587 |
| Postoperative transfusion | 1.352 (0.174~10.511) | | 0.773 |
| POD≤2days | 1.178 (0.597~2.325) | | 0.637 |

OR = Odds ratio; CI = Confidence interval. b Statistically significant (P<0.05). BMI: Body mass index; ASA: American Society of Anesthesiology; TIA: Transient cerebral ischemic attack; FEV_1_: Forced expiratory volume in 1 second; FVC: Forced vital capacity; DLCO: Diffusion capacity for carbon monoxide; RATS: Robotic-assisted thoracoscopic surgery; TPVB: Thoracic paravertebral blockade; AF: Atrial fibrillation; PPCs: Postoperative pulmonary complications; POD: Postoperative day.

Supplementary table S3A Preoperative characteristics stratified by discharge date after a 1:1 PSM

| Variables ^a^ | POD≤2days (n=1845) | | POD>2days (n=1845) | | | | SMD | *P* Value | |
| --- | --- | --- | --- | --- | --- | --- | --- | --- | --- |
| Age, years | 55.5±11.0 | | 55.4±11.3 | | | | 0.010 | 0.771 | |
| Sex |  | |  | | | | 0.019 | 0.572 | |
| Male sex | 597(32.4) | | 581(31.5) | | | |  |  | |
| Female sex | 1248(67.6) | | 1264(68.5) | | | |  |  | |
| BMI, kg/m^2^ | 23.2±2.9 | | 23.1±3.1 | | | | 0.016 | 0.633 | |
| ASA grade |  | |  | | | | 0.007 | 0.209 | |
| I | 154(8.3) | | 178(9.6) | | | |  |  | |
| II | 1497(81.1) | | 1455(78.9) | | | |  |  | |
| III/IV | 194(10.5) | | 212(11.5) | | | |  |  | |
| Comorbidity |  |  | |  |  | | | |  |
| Hypertension | 109(5.9) | | 106(5.7) | | | | 0.007 | 0.833 | |
| Diabetes mellitus | 47(2.5) | | 44(2.4) | | | | 0.010 | 0.750 | |
| Coronary artery disease | 6(0.3) | | 8(0.4) | | | | 0.018 | 0.592 | |
| Stroke/TIA | 4(0.2) | | 4(0.2) | | | <0.001 | | 1.000 | |
| FEV_1_/FVC, % | 101.9±7.3 | | 102.0±7.7 | | | | 0.011 | 0.729 | |
| DLCO% | 95.2±15.8 | | 95.3±16.2 | | | | 0.004 | 0.906 | |
| Chemoradiotherapy | 0(0.0) | | 0(0) | | | <0.001 | | 1.000 | |
| Tumor size, cm | 1.5±0.9 | | 1.5±0.9 | | | | 0.015 | 0.656 | |
| Clinical tumor stage |  | |  | | | | 0.080 | 0.433 | |
| T1a | 715(38.8) | | 701(38.0) | | | |  |  | |
| T1b | 781(42.3) | | 769(41.7) | | | |  |  | |
| T1c | 231(12.5) | | 259(14.0) | | | |  |  | |
| T2a | 71(3.8) | | 81(4.4) | | | |  |  | |
| T2b | 34(1.8) | | 25(1.4) | | | |  |  | |
| T3 | 11(0.6) | | 10(0.5) | | | |  |  | |
| T4  Advanced clinical stage (T≥2) | 2(0.1)  118(6.4) | | 0(0)  116(6.3) | | | | 0.004 | 0.893 | |
| Surgical experience < 300/y | 511(27.7) | | 520(28.2) | | | | 0.012 | 0.741 | |

^a^ Continuous data are shown as mean ± standard deviation and categoric data as number (%). b Statistically significant (P<0.05). PSM: Propensity score matching; POD: Postoperative day; SMD: Standardized mean difference; BMI: Body mass index; ASA: American Society of Anesthesiology; TIA: Transient cerebral ischemic attack; FEV_1_: Forced expiratory volume in 1 second; FVC: Forced vital capacity; DLCO: Diffusion capacity for carbon monoxide.

Supplementary table S3B Intraoperative characteristics stratified by discharge date after a 1:1 PSM

| Variables ^a^ | POD≤2days (n=1845) | POD>2days (n=1845) | | SMD | *P* Value |
| --- | --- | --- | --- | --- | --- |
| Lymph nodes calcification | 70(3.8) | 87(4.7) | | 0.046 | 0.166 |
| Clinical nodal involvement | 49(2.7) | 51(2.8) | | 0.007 | 0.839 |
| Pleural adhesions | 20(1.1) | 16(0.9) | | 0.022 | 0.503 |
| Type of resection |  |  | | 0.012 | 0.705 |
| Segmentectomy resection | 657(35.6) | 646(35.0) | |  |  |
| Lobectomy resection | 1188(64.4) | 1199(65.0) | |  |  |
| Procedure |  |  | | 0.008 | 0.809 |
| Uni-portal | 243(13.2) | 248(13.4) | |  |  |
| Multi-portal | 1602(86.8) | 1845(86.6) | |  |  |
| Approach |  |  | | 0.030 | 0.363 |
| VATS | 1820(98.6) | 1826(99.0) | |  |  |
| RATS | 25(1.4) | 19(1.0) | |  |  |
| Anesthesia type |  |  | | 0.010 | 0.757 |
| General anesthesia | 1535(83.2) | 1542(83.6) | |  |  |
| Combined with TPVB | 310(16.8) | 303(16.4) | |  |  |
| Location of resection |  |  | | 0.028 | 0.397 |
| Left resection | 693(37.6) | 718(38.9) | |  |  |
| Right resection | 1152(62.4) | 1127(61.1) | |  |  |
| Ipsilateral reoperation | 1(0.1) | 1(0.1) | <0.001 | | 1.000 |
| Conversion to thoracotomy | 8(0.4) | 11(0.6) | | 0.023 | 0.490 |
| Intraoperative transfusion | 0(0) | 0(0) | <0.001 | | 1.000 |
| Intraoperative new onset AF | 16(0.9) | 18(1.0) | | 0.011 | 0.730 |
| Operative time, mins | 86.4±32.3 | 86.6±28.8 | | 0.007 | 0.841 |

^a^ Continuous data are shown as mean ± standard deviation and categoric data as number (%).

b Statistically significant (P<0.05). PSM: Propensity score matching; POD: Postoperative day; SMD: Standardized mean difference; VATS: Video-assisted thoracoscopic surgery; RATS: Robotic-assisted thoracoscopic surgery; TPVB: Thoracic paravertebral blockade; AF: Atrial fibrillation.

Supplementary table S4 Multivariable **logistic regression analysis** of predictors for 30-day readmission after a 1:1 PSM

| Variables | OR (95% CI) | *P* Value |
| --- | --- | --- |
| Age, years | 1.017 (0.979~1.056) | 0.384 |
| Male sex | 1.023 (0.446~2.345) | 0.957 |
| BMI, kg/m^2^ | 0.965 (0.840~1.107) | 0.608 |
| ASA grade |  |  |
| I(reference) |  |  |
| II | 0.154 (0.063~0.377) | <0.001^b^ |
| III/IV | 0.216 (0.053~0.871) | 0.031^b^ |
| Hypertension | 1.537 (0.347~6.808) | 0.571 |
| FEV_1_/FVC, % | 0.981 (0.939~1.024) | 0.374 |
| DLCO% | 0.996 (0.974~1.019) | 0.742 |
| Tumor size, cm | 1.435 (0.837~2.461) | 0.189 |
| Advanced clinical stage (T≥2) | 0.476 (0.074~3.086) | 0.437 |
| Surgical experience < 300/y | 0.907 (0.393~2.095) | 0.819 |
| Lymph nodes calcification | 2.345 (0.686~8.020) | 0.174 |
| Clinical nodal involvement | 1.117 (0.215~5.801) | 0.895 |
| Lobectomy resection | 2.115 (0.798~5.609) | 0.132 |
| Multi-portal | 1.199 (0.393~3.660) | 0.750 |
| RATS | 0.758 (0.416~1.380) | 0.364 |
| Combined with TPVB | 1.384 (0.496~3.864) | 0.535 |
| Right resection | 0.638 (0.297~1.372) | 0.250 |
| Operative time, mins | 1.005 (0.992~1.017) | 0.458 |
| PPCs  Postoperative transfusion | 1.100 (0.384~3.147)  9.852(0.921~10.380) | 0.859  0.059 |
| POD≤2days | 0.977(0.409~2.332) | 0.958 |

OR = Odds ratio; CI = Confidence interval. b Statistically significant (P<0.05). BMI: Body mass index; ASA: American Society of Anesthesiology; TIA: Transient cerebral ischemic attack; FEV_1_: Forced expiratory volume in 1 second; FVC: Forced vital capacity; DLCO: Diffusion capacity for carbon monoxide; TPVB: Thoracic paravertebral blockade; AF: Atrial fibrillation; PPCs: Postoperative pulmonary complications; POD: Postoperative day.
